# Microsurgery of Skull Base Paraganglioma

**Published:** 2014-01

**Authors:** Seb Thomas

Skull base paragangliomas are rare tumours and there are few specific texts on the subject. This book brings together a huge amount of clinical and surgical information on a wide variety of cases. Professor Sanna and his team at the Gruppo Otologico of Italy must be congratulated for achieving this.

The first seven chapters of the book describe the genetics, surgical anatomy, radiology, preoperative investigation and decision making. These chapters are illustrated superbly with cadaveric dissections, clinical photographs and scans as well as high quality diagrams, text boxes and flowcharts.

The subsequent 13 chapters deal with the surgical management of head and neck paragangliomas at different sites and different stages. In addition, there are chapters addressing the surgical management of specific problems including the involved internal carotid artery, facial nerve, lower cranial nerves and vertebral artery.

These latter chapters tend to be laid out in two parts. The initial written section describes the surgical approach, illustrated with cadaveric dissections and diagrams. The second section describes one or more typical clinical cases and their operations. Here, preoperative clinical and radiological photographs of the particular case are shown followed by multiple high quality operative photographs depicting the important steps of the operation used to manage the case.

The book is written in a ‘how we do it’ style. In places, the discussion of controversial areas is limited. However, surgical procedures are described and illustrated with much clarity. The great strength of this book is in its multiple high quality intraoperative and cadaveric photographs used to illustrate the text. These, together with the problem centred approach of the volume, will be of much interest to all surgeons working in the field.

